# Trends in platelet count among cancer patients

**DOI:** 10.1186/s40164-022-00272-3

**Published:** 2022-03-24

**Authors:** Vasily Giannakeas

**Affiliations:** 1 Women’s College Research Institute, Women’s College Hospital, 76 Grenville Street, 6th Floor, Toronto, ON M5S 1B2 Canada; 2 Dalla Lana School of Public Health, University of Toronto, 155 College Street, Health Science Building, 6th Floor, Toronto, ON Canada; 3 ICES, Toronto, ON Canada

**Keywords:** Platelets, Platelet count, Cancer incidence, Cancer survival

## Abstract

An elevated platelet count has been associated with an increased incidence of cancer and poor survival for many cancer types. In this study, platelet levels were captured among cancer patients in the 2 years prior to and following a cancer diagnosis. I investigated if the trends in platelet count differ between patients that died or did not die from their cancer. For many cancer types, including colon, lung, ovary, and stomach, platelet counts rose as they approached the date of diagnosis. Patients that died from their cancer within 3 years of diagnosis had a higher peak platelet count than those who survived. Following diagnosis, platelet count was elevated among patients that died from their cancer as compared to patients who survived. An elevated platelet count could potentially indicate the presence of an occult cancer or be used as a prognostic measure for cancer-specific survival.

**Supplementary Information:**

The online version contains supplementary material available at 10.1186/s40164-022-00272-3.

To the Editor,

It is known that platelets play a key role in many stages of the natural history of cancer, from tumour growth to cancer dissemination and metastasis [1, 2]. Thrombocytosis is associated with an increased incidence of several cancers [3]. Moreover, thrombocytosis is associated with poor cancer-specific survival. The associations are particularly strong for ovarian cancer—an elevated platelet count is associated with a 7-fold increased risk of ovarian cancer [4] and a 1.7-fold increased risk of death among ovarian cancer patients [5]. Others have shown that an elevated platelet count accelerates ovarian cancer progression [6].

It remains unclear to what extent the trajectory of platelet counts (prior to and following a diagnosis) differs for cancer patients who survive their cancer, versus patients that succumb to their disease. Using electronic medical records from the province of Ontario, Canada, I identified provincial residents with a first cancer diagnosis between January 2007 and December 2015. Study subjects were patients with at least one complete blood count (CBC) record in the two-year period preceding or following a cancer diagnosis of the colon, lung, breast, prostate, stomach, or ovary. The study cohort consisted of 213,336 cancer patients of whom 59,519 (27.9%) died from their cancer in the period following diagnosis. The mean age at diagnosis was 66.7 years and 107,754 (50.5%) of the patients were female. In total there were 1,700,764 CBC records in the 4-year observation period; the mean number of CBC records in the two years prior to diagnosis was 2.0, and the mean number of CBC records in the 2 years after diagnosis was 6.0.

For each cancer site, I divided the cohort into survivors and non-survivors by assigning patients to two strata; those who died from their cancer within 3 years of diagnosis, and those who did not die from their cancer in the 3 years after diagnosis. Median platelet count was measured in biweekly intervals for 2 years before and after diagnosis, for each stratum.

 I first analyzed the data on ovarian cancer. Descriptive information on the ovarian cancer cohort is presented in Table 1. Median platelet count is plotted for the period from 2 years before diagnosis to 2 years after diagnosis (Fig. 1). For ovarian cancer patients, there is a slow rise in platelet count for the first 18 months and then a rapid rise in the 6 months prior to diagnosis, when the two curves begin to split (Fig. 1). At diagnosis, patients who died from ovarian cancer have a higher median platelet count than the patients who survived. After diagnosis, platelet counts for both groups dropped precipitously to below pre-diagnostic levels by 6 months. From month 6 to month 12 they rose again—but the rise was much steeper for those who died than for those who survived 3 years.

**Table 1:** Characteristics of the ovarian cancer patient cohort measured at the diagnosis date

Description	Value	
Number of patients		6451
Year of diagnosis	Mean (SD)	2011.5 (2.4)
	Median (IQR)	2012 (2009-2014)
Age	Mean (SD)	62.2 (14.9)
	Median (IQR)	62.6 (52.0–72.9)
Residence setting	Urban	5733 (88.9%)
	Rural	712 (11.0%)
	Missing	6 (0.1%)
Primary care visits to general practitioner in previous 2 years	Mean (SD)	3.1 (3.3)
	Median (IQR)	2 (1–4)
Comorbidities	Asthma	582 (9.0%)
	Congestive heart failure	301 (4.7%)
	Hypertension	2922 (45.3%)
	Diabetes	806 (12.5%)
	Dementia	164 (2.5%)
Number of concurrent medications used	Mean (SD)	3.9 (3.1)
	Median (IQR)	3 (2-6)
Cancer stage	I	1140 (17.7%)
	II	496 (7.7%)
	III	2189 (33.9%)
	IV	801 (12.4%)
	Unknown	1825 (28.3%)
Number of CBCs during the observation period	Mean (SD)	12.0 (16.0)
	Median (IQR)	6 (2–15)
Number of CBCs in the 2 years prior to diagnosis	Mean (SD)	2.0 (2.8)
	Median (IQR)	1 (0–3)
Number of CBCs in the 2 years after diagnosis	Mean (SD)	10.0 (15.4)
	Median (IQR)	4 (1–13)
Ovarian cancer death within 3 years of diagnosis	Yes	2302 (35.7%)

Similar trends were seen for colon cancer, lung cancer, and stomach cancer, for both female and male patients (Additional file 1: Fig. S1, S2). We have previously shown that a high platelet count is associated with an increased incidence and worse survival for these cancers [4, 7]. In contrast, for breast and prostate cancer, the separation was minimal before diagnosis but not after diagnosis (Additional file 1: Fig. S1, S2). These findings are also consistent with our previous work, which showed no association between an elevated platelet count and cancer incidence for breast and prostate cancer [4], but worse cancer-specific survival among patients with an elevated platelet count [7]. The basis for the variation in platelet count between cancer survivors and those who succumb to cancer is not known but merits further investigation.

**Fig. 1:**
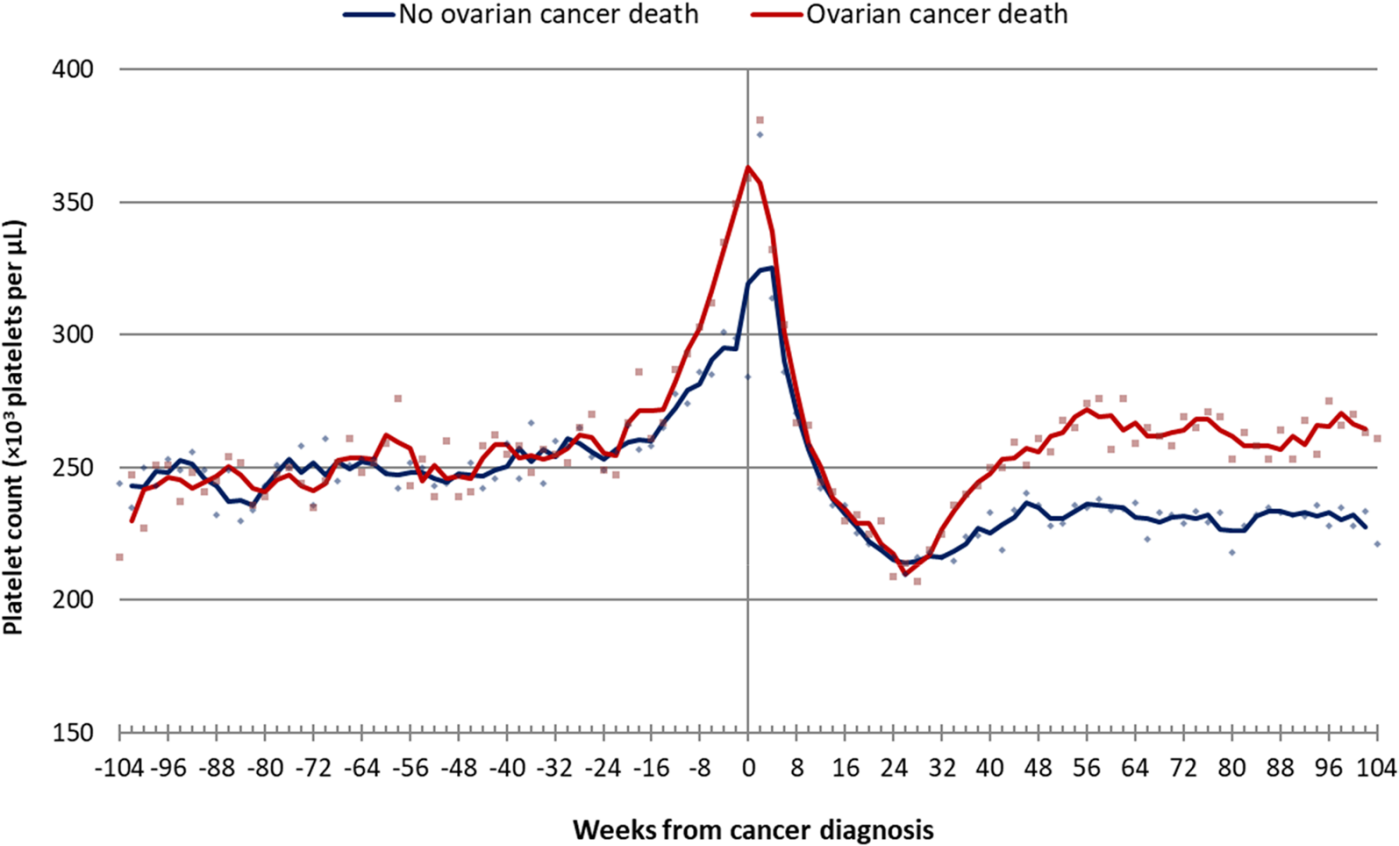
Median platelet count measured biweekly (and 3 period moving average) among ovarian cancer patients that died from their cancer within 3 years, versus all other ovarian cancer patients

## Supplementary Information

**Additional file 1: Table S1.** Characteristics of cancer patient cohort. **Figure S1. **Median platelet count measured biweekly (and 3 period moving averages) among female patients diagnosed with (A) breast cancer, (B) colon cancer, (C) lung cancer, and (D) stomach cancer. **Figure S2. **Median platelet count measured biweekly (and 3 period moving averages) among male patients diagnosed with (A) prostate cancer, (B) colon cancer, (C) lung cancer, and (D)stomach cancer.

## Data Availability

VG had full access to all the data in the study and takes responsibility for the integrity of the data and the accuracy of the data analysis.
