# Supplementary material for: Trends in platelet count among cancer patients
Source: Exp Hematol Oncol. 2022 Mar 24;11:16. doi: 10.1186/s40164-022-00272-3 (PMC8944120; doi:10.1186/s40164-022-00272-3)

**Additional file 1**

**Table S1.**

Characteristics of cancer patient cohort.

|  |  |  | **Cancer-specific death within 3 years of diagnosis** | |
| --- | --- | --- | --- | --- |
| **Variable** | **Value** | **Total** | **No** | **Yes** |
| *Overall* |  | *213,336* | *165,348 (77.5%)* | *47,988 (22.5%)* |
| Year of diagnosis | Mean (SD) | 2011.5 (2.4) | 2011.4 (2.5) | 2011.7 (2.3) |
|  | Median (IQR) | 2012.(2010-2014) | 2012 (2009-2014) | 2012 (2010-2014) |
| Sex | Female | 107,754 (50.5%) | 83,620 (50.6%) | 24,134 (50.3%) |
|  | Male | 105,582 (49.5%) | 81,728 (49.4%) | 23,854 (49.7%) |
| Age at diagnosis | Mean (SD) | 66.7 (12.6) | 65.5 (12.3) | 71.1 (12.5) |
|  | Median (IQR) | 67.2 (58.4-75.8) | 66.0 (57.4-74.1) | 72.2 (62.8-80.7) |
| Cancer type | Breast | 57,012 (26.7%) | 53,472 (32.3%) | 3,540 (7.4%) |
|  | Prostate | 55,567 (26.0%) | 52,833 (32.0%) | 2,734 (5.7%) |
|  | Colon | 45,461 (21.3%) | 35,506 (21.5%) | 9,955 (20.7%) |
|  | Lung | 41,727 (19.6%) | 16,107 (9.7%) | 25,620 (53.4%) |
|  | Stomach | 7,118 (3.3%) | 3,281 (2.0%) | 3,837 (8.0%) |
|  | Ovary | 6,451 (3.0%) | 4,149 (2.5%) | 2,302 (4.8%) |
| Number of CBCs during the observation period | Mean (SD) | 8.0 (11.3) | 7.5 (10.3) | 9.6 (14.0) |
|  | Median (IQR) | 4 (2-9) | 4 (2-9) | 4 (2-11) |
| Number of CBCs in the 2 years prior to diagnosis | Mean (SD) | 2.0 (3.3) | 1.8 (3.0) | 2.6 (4.0) |
|  | Median (IQR) | 1 (0-2) | 1 (0-2) | 2 (1-3) |
| Number of CBCs in the 2 years after diagnosis | Mean (SD) | 6.0 (10.2) | 5.7 (9.2) | 7.0 (13.2) |
|  | Median (IQR) | 2 (1-6) | 3 (1-6) | 1 (0-8) |
| Follow-up time (years) | Mean (SD) | 4.9 (3.2) | 6.1 (2.6) | 0.9 (0.8) |
|  | Median (IQR) | 4.8 (2.2-7.6) | 5.9 (4.1-8.3) | 0.7 (0.2-1.5) |
| Cancer-specific death at any point in follow-up period | No | 153,817 (72.1%) | 153,817 (93.0%) | 0 (0.0%) |
|  | Yes | 59,519 (27.9%) | 11,531 (7.0%) | 47,988 (100.0%) |

**Figure S1.**

Median platelet count measured biweekly (and 3 period moving averages) among female

patients diagnosed with (A) breast cancer, (B) colon cancer, (C) lung cancer, and

(D) stomach cancer.

**(A)** **Breast cancer patients**

**
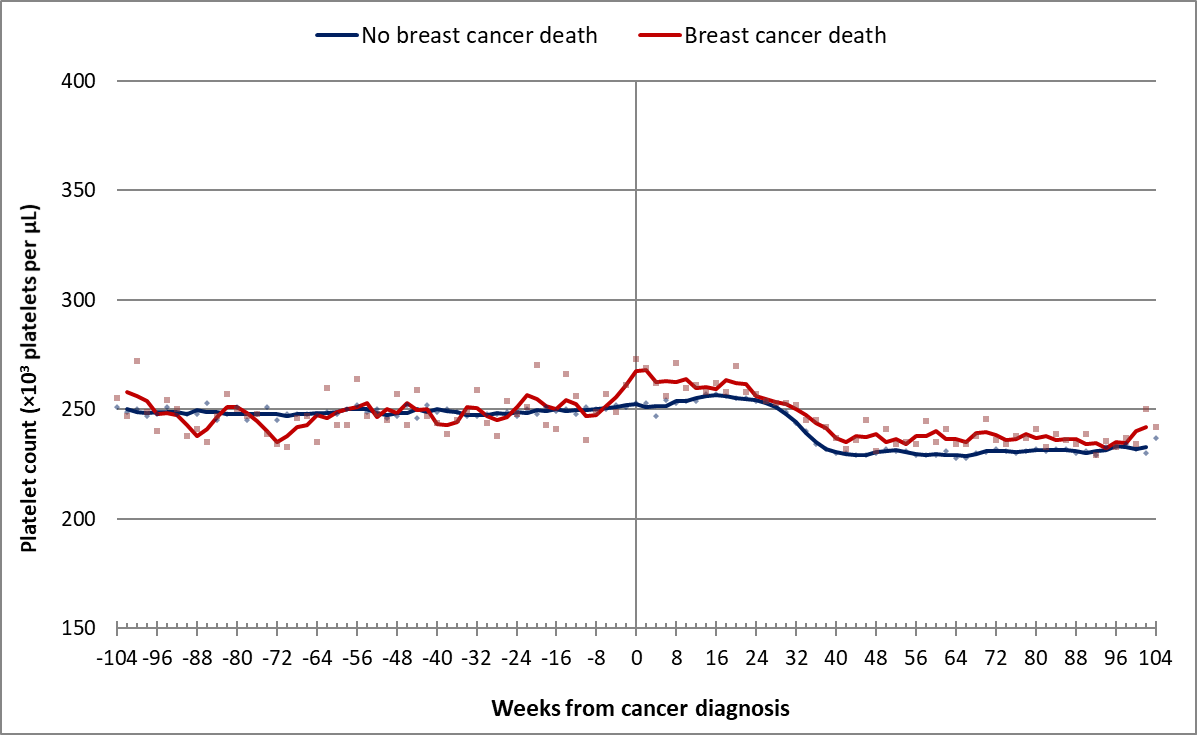
**

**(B) Colon cancer patients**


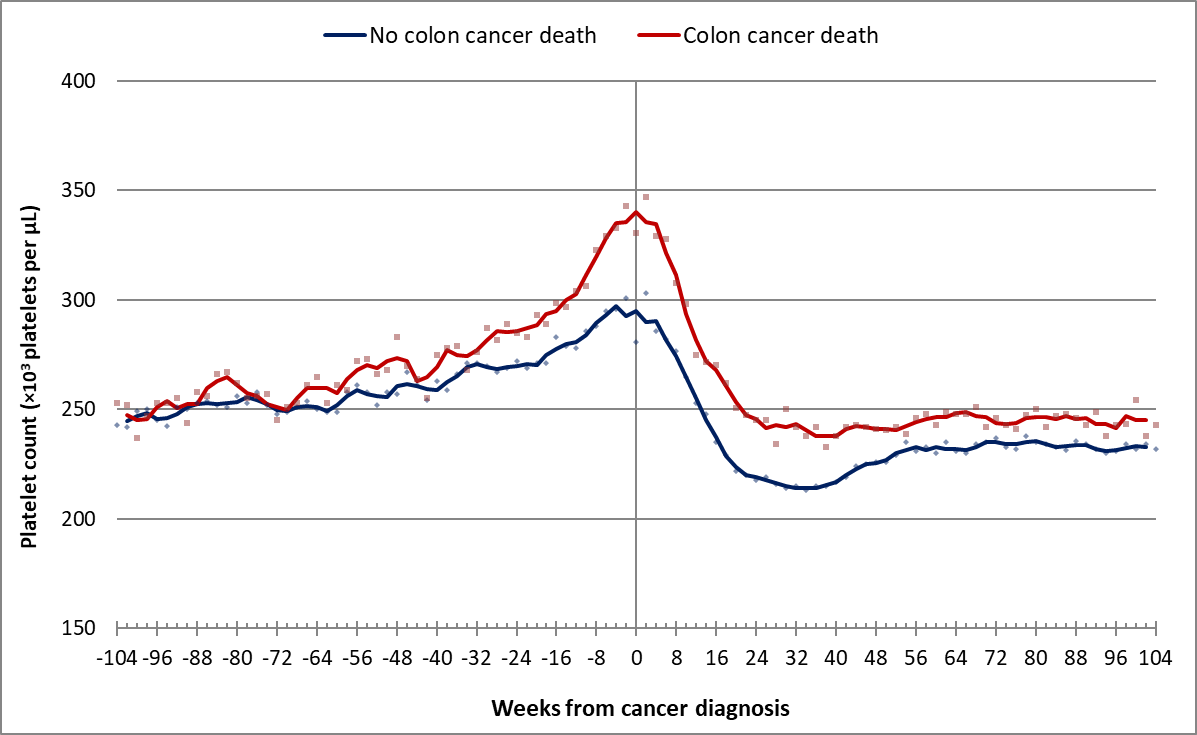


**(C) Lung cancer patients**


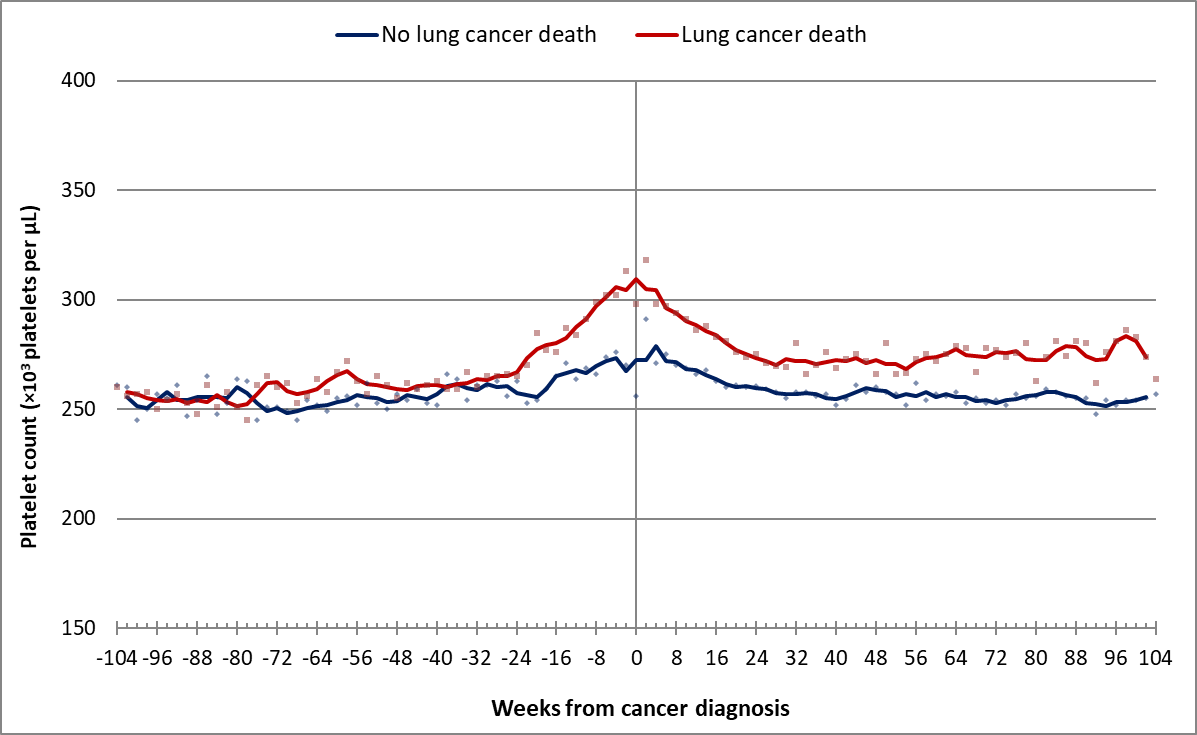


**(D)** **Stomach cancer patients**


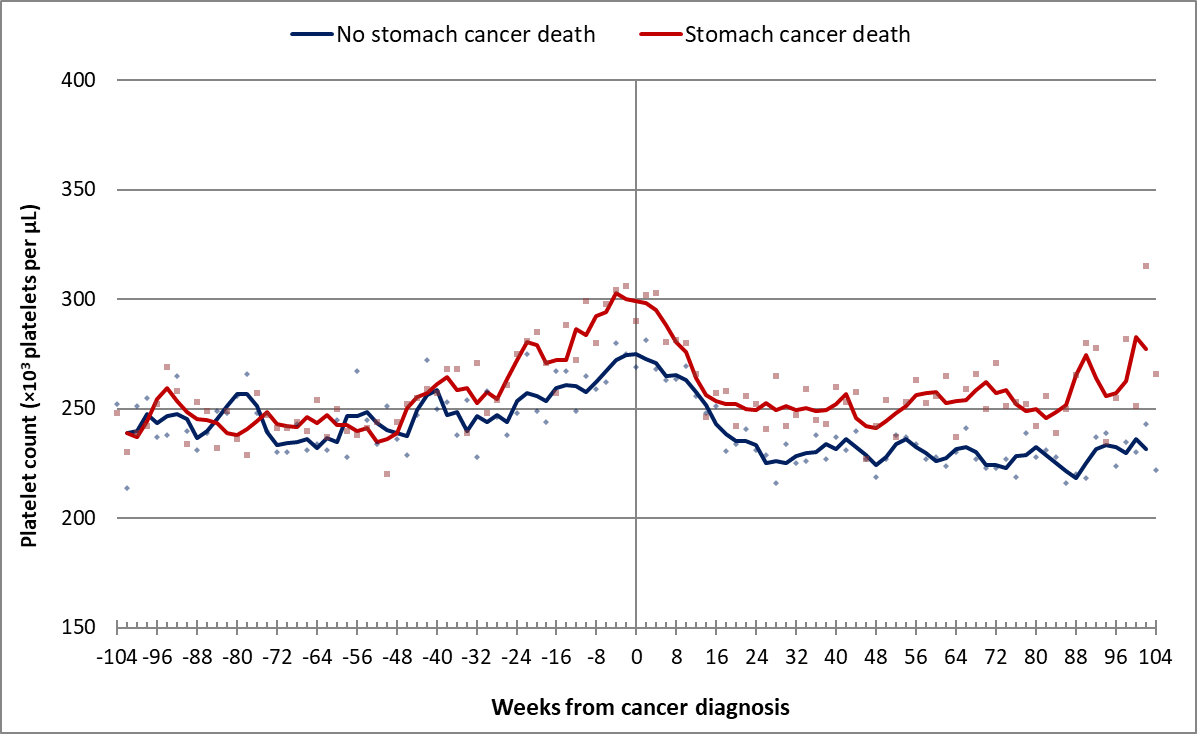


**Figure S2.**

Median platelet count measured biweekly (and 3 period moving averages) among male

patients diagnosed with (A) prostate cancer, (B) colon cancer, (C) lung cancer, and

(D) stomach cancer.

**(A)** **Prostate cancer patients**

**
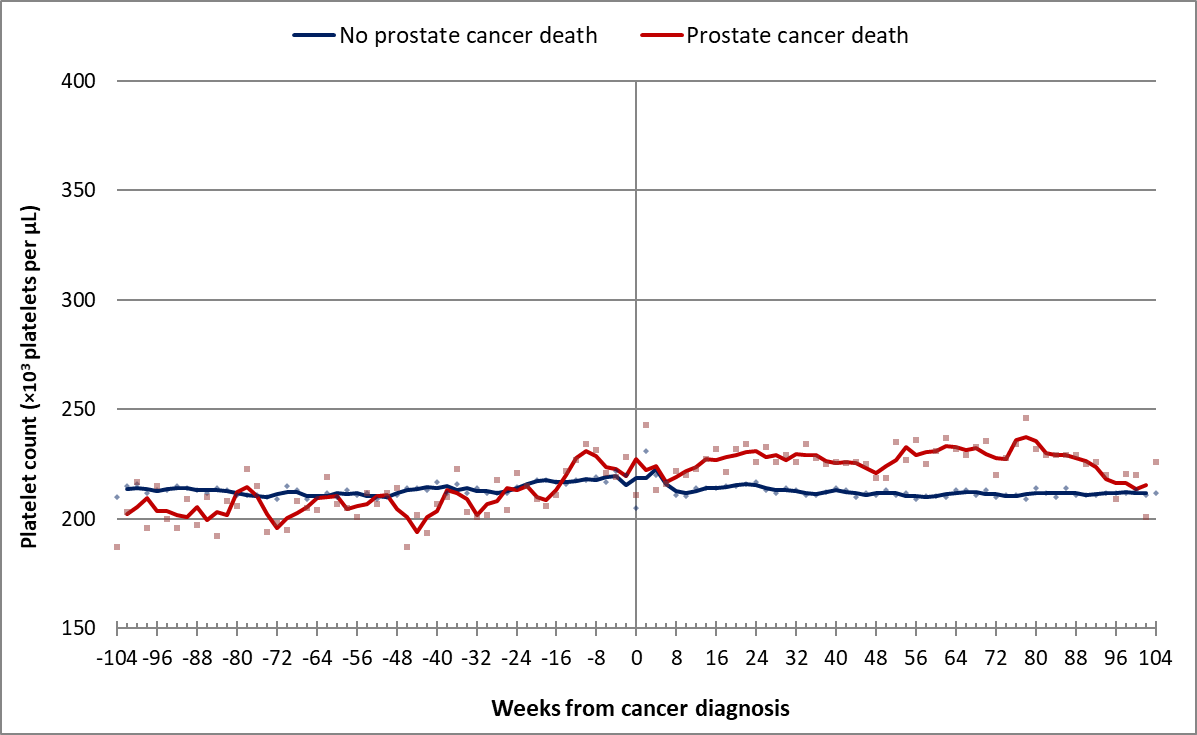
**

**(B) Colon cancer patients**


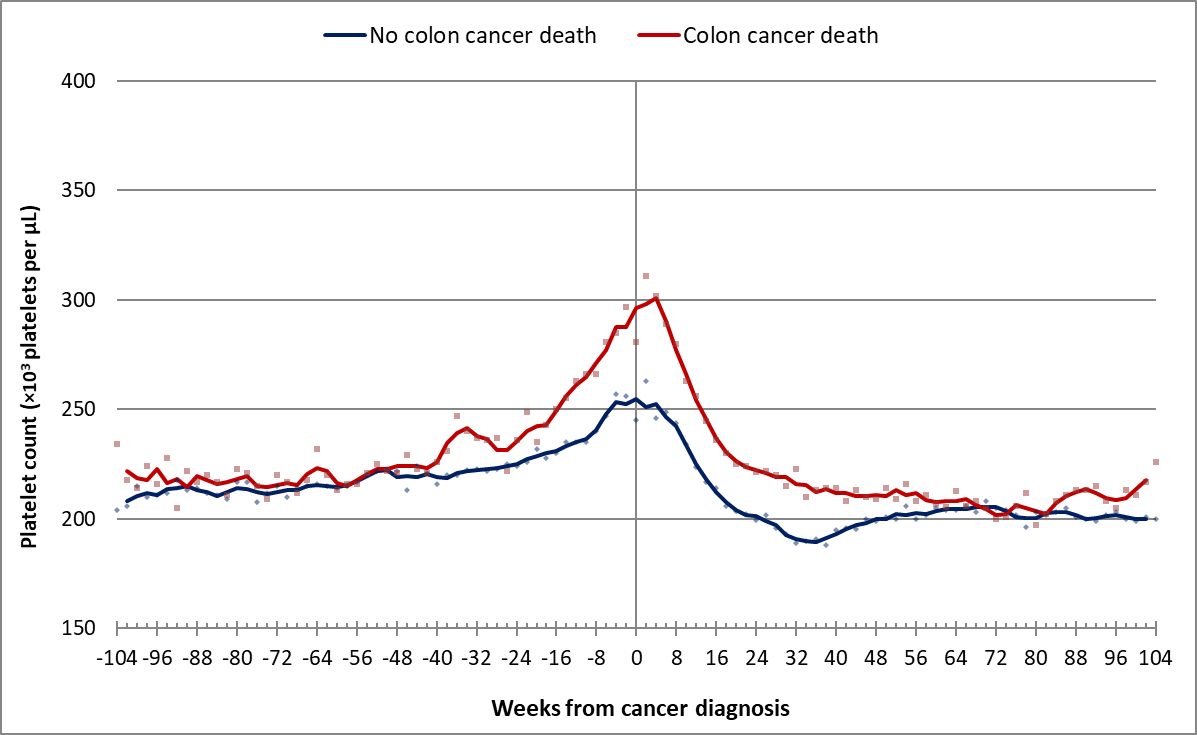


**(C) Lung cancer patients**


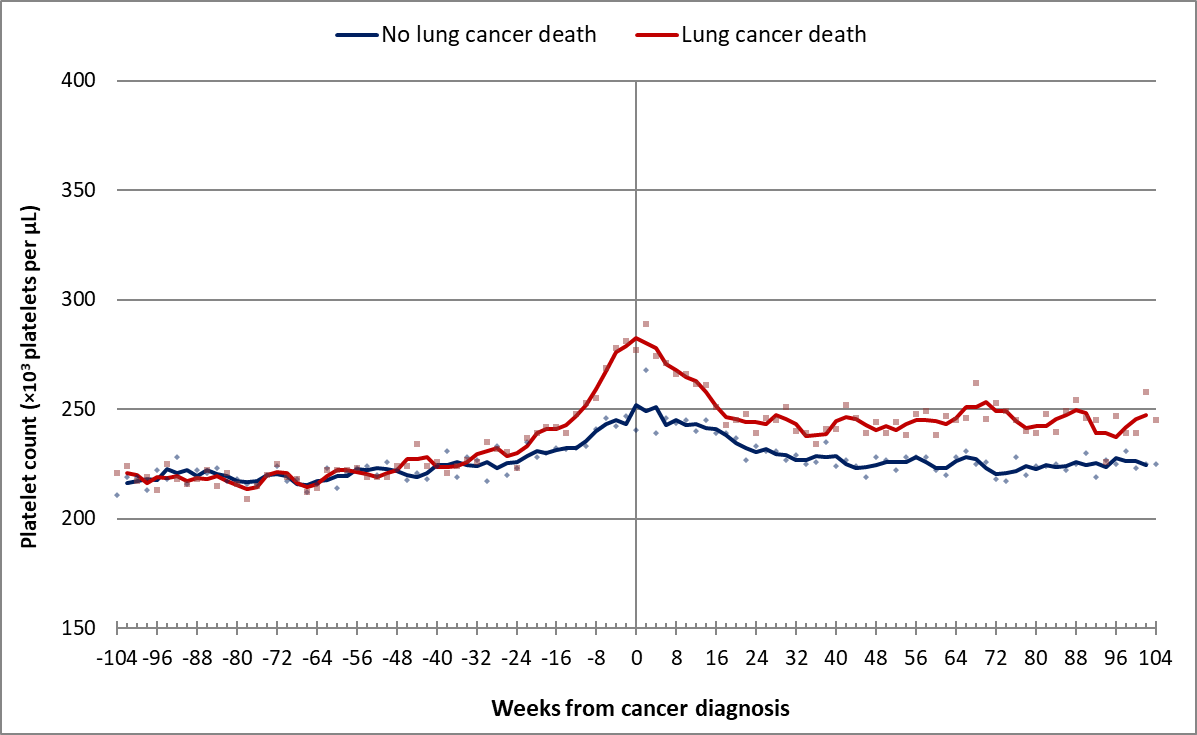


**(D)** **Stomach cancer patients**


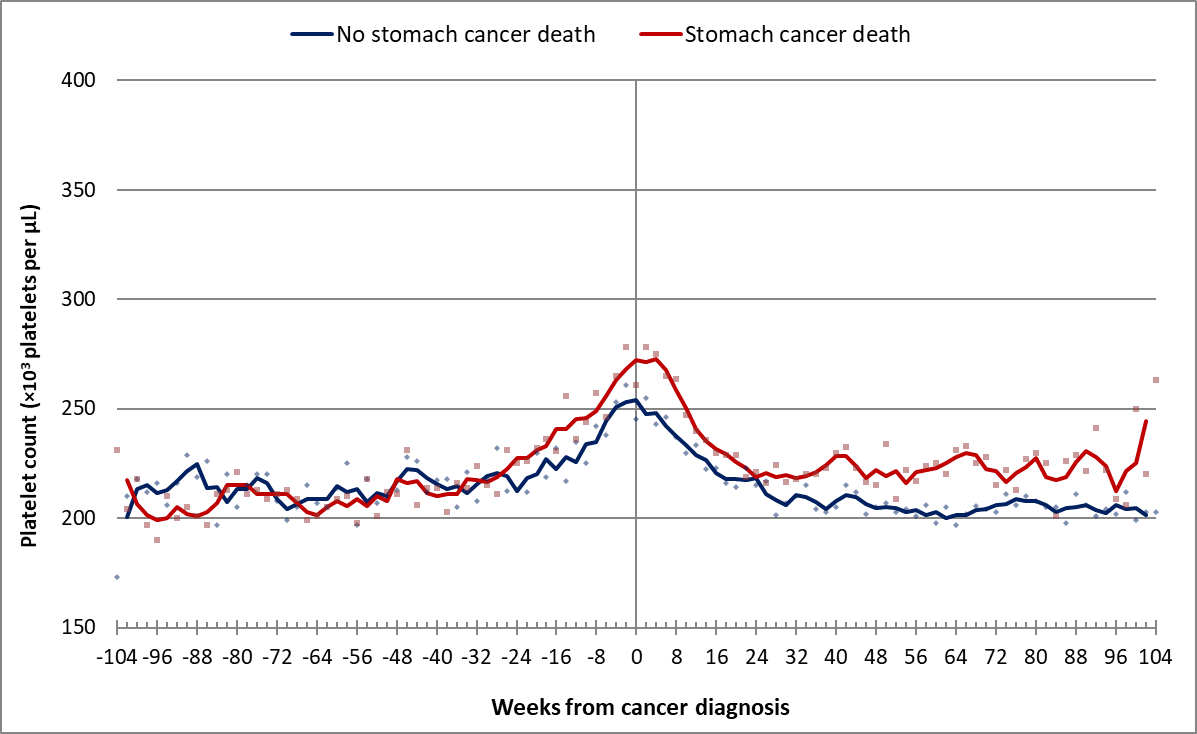

Supplement: Supplementary file 1 — Additional file 1: Table S1. Characteristics of cancer patient cohort. Figure S1. Median platelet count measured biweekly (and 3 period moving averages) among female patients diagnosed with (A) breast cancer, (B) colon cancer, (C) lung cancer, and (D) stomach cancer. Figure S2. Median platelet count measured biweekly (and 3 period moving averages) among male patients diagnosed with (A) prostate cancer, (B) colon cancer, (C) lung cancer, and (D)stomach cancer. [file 40164_2022_272_MOESM1_ESM.docx]
